# Molecular investigation of *Neospora caninum* in cattle in the Khomas region of Namibia

**DOI:** 10.4102/ojvr.v92i1.2237

**Published:** 2025-11-14

**Authors:** Alaster Samkange, Simbarashe Chitanga, Pricilla Mbiri, Ophelia C. Matomola, Luis Neves, Paul T. Matjila

**Affiliations:** 1 Department of Veterinary Tropical Diseases, Faculty of Veterinary Science, University of Pretoria, Pretoria, South Africa; 2 Department of Production Animal Clinical Studies, Faculty of Health Sciences and Veterinary Medicine, University of Namibia, Windhoek, Namibia; 3 Department of Pre-clinical Studies, Faculty of Health Sciences and Veterinary Medicine, University of Namibia, Windhoek, Namibia; 4 Department of Biomedical Sciences, Faculty of Health Sciences, University of Zambia, Lusaka, Zambia; 5 School of Life Sciences, College of Agriculture, Engineering and Sciences, University of KwaZulu-Natal, Durban, South Africa; 6 Centro de Biotecnologia, Universidade Eduardo Mondlane, Maputo, Mozambique

**Keywords:** *Neospora*, molecular, detection, cattle, Khomas, region, Namibia

## Abstract

Neosporosis is a leading cause of bovine abortions worldwide, with a substantial economic impact because of reproductive losses, costing the cattle industry billions of dollars annually. Despite the global significance of *Neospora caninum*, molecular studies on this protozoan parasite have yet to be conducted in cattle in Southern Africa, leaving a critical knowledge gap in countries like Namibia, where beef production is vital to the economy. This study aimed to detect latent *N. caninum* infections in beef cattle brain and heart muscle samples collected from abattoirs and blood samples from *Neospora*-seropositive animals within Namibia’s Khomas region. One hundred and ninety-nine samples were collected, comprising 110 brain samples, 75 heart muscle samples and 14 whole blood samples. Conventional polymerase chain reaction (PCR) analysis was performed using Np6 and Np21 primers targeting a ≈340 base pair (bp) segment of the Nc5 gene. All samples tested negative. These results were likely because of the low prevalence of this parasite in Namibian beef cattle, as reported in a previous serosurvey. Future studies focusing on aborted foetuses over a longer timeframe may increase the likelihood of detecting positive cases.

**Contribution:**

This study represents the first published molecular investigation of *N. caninum* in beef cattle in Southern Africa. The negative results underscore the challenges of detecting *N. caninum* in tissues from clinically healthy cattle, particularly in a semi-arid country like Namibia, where the prevalence of the pathogen is inherently low.

## Introduction

Bovine neosporosis, caused by *Neospora caninum*, is a major cattle disease characterised by abortion, stillbirths, mummified or resorbed foetuses, weak calves and infertility; no effective treatment or vaccine exists (Al-Qassab, Reichel & Ellis 2010; Pereira et al. 2022). Infected cows may also give birth to seemingly healthy but persistently infected calves capable of vertical transmission (Al-Qassab et al. 2010; Müller et al. 2015). Globally, the disease causes annual losses exceeding $1 billion (Reichel et al. 2013).

Canids, including dogs, wolves, dingoes and coyotes, are the definitive hosts, while cattle, sheep, goats, birds and other herbivores serve as intermediate hosts (Al-Qassab et al. 2010). *Neospora caninum* has three infective stages: tachyzoites, bradyzoites in tissue cysts and sporozoites formed in the definitive host’s intestine (Müller et al. 2015). Tachyzoites mediate acute *N. caninum* infection, emerging after sporozoite invasion of intestinal epithelium and rapidly disseminating to host tissues, where they multiply via lytic cycles (Donahoe et al. 2015). In contrast, bradyzoites form under immune pressure – driven by gamma interferon – and establish chronic infection through slow replication within cysts in neural and muscular tissues (Al-Qassab et al. 2010; Fereig & Nishikawa 2020; Marugan-Hernandez 2017). The parasite primarily localises in the brain of intermediate hosts, although deoxyribonucleic acid (DNA) has been detected in the placenta, blood, serum, milk, colostrum and semen (Gharekhani, Yakhchali & Heidari 2022). In cattle, it has been found in the brain (amygdala and hippocampus), spinal cord, heart, lung, diaphragm and skeletal muscle (Nishimura et al. 2013).

Molecular studies report variable prevalence across species and regions: 2.8% in goat brains in China (Qian et al. 2020); 13.6% in cattle and 35.4% in aborted foetuses in Iran (Gharekhani et al. 2022); 22% in cattle and 10.6% in ewes in Tunisia (Amdouni et al. 2018); and no detection in South African birds (Lukášová et al. 2018). In Egypt, *N. caninum* DNA was found in bovine milk (Fereig et al. 2022) and in Italy, genetic clustering correlated with geographic origin (Villa et al. 2021).

Despite this, molecular data on *N. caninum* in sub-Saharan Africa are scarce, particularly in Namibia. This study aimed to investigate the presence of *N. caninum* DNA in cattle brains, heart muscles and blood samples from seropositive animals in the Khomas region of Namibia, representing the first molecular investigation of the parasite in cattle in Southern Africa.

## Research methods and design

### Study population

The study population included clinically healthy cattle slaughtered at four abattoirs in Namibia’s Khomas region, near the capital, Windhoek. Blood samples were also collected from a subset of *N. caninum* seropositive animals from farms previously studied (Samkange 2023; Samkange et al. 2023). Abattoir throughput varied, with some processing 1–10 cattle weekly and others over 100. All animals underwent ante-mortem and post-mortem inspections by veterinarians or environmental health practitioners, depending on the facility.

### Sample collection

Tissue samples of approximately 2 cm^2^ were collected from the heart muscle and brain tissue of cattle carcasses at four abattoirs. Additionally, 7 mL whole blood samples in ethylenediaminetetraacetic acid (EDTA) were taken from seropositive animals on farms previously studied (Samkange 2023; Samkange et al. 2023). A total of 199 samples were collected from different animals: 110 brain, 75 heart, and 14 blood samples. Sterilised instruments were used to collect tissues; brain samples were scooped through the foramen magnum using a plastic spoon. Blood was drawn from the coccygeal vein with a 20-gauge needle into EDTA tubes. All tools were sterilised between uses. Samples were collected from July 2022 to February 2023 and stored at –20 °C before analysis.

### Deoxyribonucleic acid extraction

Genomic DNA was extracted from blood, brain and heart samples using the Quick-DNA™ Miniprep Plus Kit (Zymo Research, United States [US]), following the manufacturer’s instructions. Samples (≤ 25 mg) were digested with Proteinase K (20 mg/mL) in Solid Tissue Buffer at 55 °C overnight. After centrifugation, lysates were processed through a spin column, washed and eluted in 50 µL of elution buffer, then stored at –20 °C. High-quality DNA was successfully extracted, and concentrations were measured using the Qubit 4 Fluorometer (Thermo Fisher Scientific), yielding values between 10 ng/µL and 100 ng/µL.

### Polymerase chain reaction

A conventional polymerase chain reaction (PCR) targeting the Nc5 gene (≈340 base pairs [bp]) was conducted using primers Np6 (forward: 5’-CAGTCAACCTACGTCTTC-3’) and Np21 (reverse: 5’-GTGCGTCCAATCCTGTAA-3’) (Karakavuk et al. 2021), using an Eppendorf Flexlid^®^ thermocycler. The PCR reaction was conducted using a 12.5 µL total mixture consisting of 2 µL of genomic DNA, 6.2 µL of One Taq^®^ master mix (containing Taq DNA polymerase, dNTPs, MgCl2 and a buffer), 1.2 µL of each primer (10 pmol/µL starting concentration) (Inqaba Biotec, Pretoria, South Africa), and 1.9 µL of nuclease-free water. Amplification conditions included initial denaturation at 95 °C for 2 min, followed by 35 cycles of 95 °C (15 s), 46 °C (15 s), 72 °C (30 s) and final extension at 72 °C for 5 min. A positive control consisting of *N. caninum* DNA (Field strain, Italy) and a negative control containing nuclease-free water were incorporated into each reaction. Polymerase chain reaction products were resolved on 1.8% agarose gels stained with 0.05% ethidium bromide, and visualised under ultraviolet (UV) light.

### Ethical considerations

Ethical clearance to conduct this study was obtained from the University of Namibia Ethics Committee (No. NEC0007), the University of Pretoria Animal Ethics Committee (No. REC087-21) and the University of Pretoria Research Ethics Committee (No. HUM005/0322). Consent was obtained from all participating abattoirs.

## Results

**FIGURE 1:**
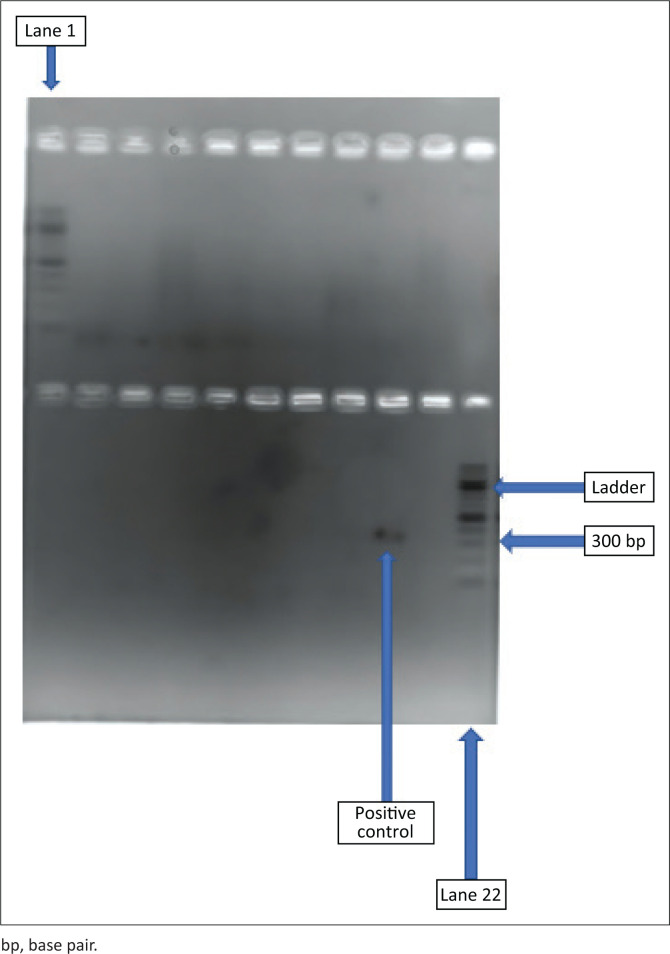
Picture showing the gel electrophoresis of polymerase chain reaction products after 20 min. Lanes 1 and 22 were laden with a 100 base pair ladder (Inqaba Biotec, South Africa). Lanes 20 and 21 were positive and negative controls, respectively. Lanes 2–19 had sample polymerase chain reaction products and are all negative.

Conventional PCR assays yielded negative results for all 199 samples, including 110 brain samples and 75 heart muscle samples from four abattoirs, as well as 14 whole blood samples from three farms (Table 1 & Figure 1). The 14 blood samples tested were all from pregnant cows that were seropositive on enzyme-linked immunosorbent assay (ELISA) from an earlier study (Samkange 2023; Samkange et al. 2023), but *Neospora* DNA could not be detected on PCR.

**TABLE 1:** Summary of the polymerase chain reaction results according to locality and type of deoxyribonucleic acid samples tested.

Locality	Hearts	Brain	Whole blood
Abattoir 1	2	2	0
Abattoir 2	33	69	0
Abattoir 3	13	13	0
Abattoir 4	27	26	0
Farm 1	0	0	7
Farm 2	0	0	6
Farm 3	0	0	1
Totals	75	110	14
Number positive	0	0	0

## Discussion

This study followed a previous serosurvey in the Khomas region, which reported a low animal-level *N. caninum* seroprevalence of 5.7% (*n* = 736), compared to rates in other countries (Samkange et al. 2023). The failure to detect *N. caninum* DNA in all 199 samples is likely a reflection of the low infection prevalence, possibly coupled with low parasitaemia in chronically infected cattle, which limits detection by conventional methods. Larger sample sizes or targeting high-risk groups, such as animals with a history of abortion or aborted foetuses, could improve detection rates (Nayeri et al. 2022). For example, molecular prevalence in aborted bovine foetuses has been reported as high as 20.5% in Iran and 22% in Tunisia (Amdouni et al. 2018; Salehi et al. 2021).

Namibia’s semi-arid to arid climate, particularly in central regions, features extreme heat and low humidity, conditions known to rapidly degrade coccidian oocysts. Studies show that at temperatures above 25 °C and low relative humidity, oocysts lose viability within 24 h (Langkjær & Roepstorff 2008). These harsh conditions likely inhibit oocyst survival and sporulation, reducing environmental contamination and transmission risk to intermediate hosts.

Sampling limitations could also have contributed to negative results. Brain samples were collected via the foramen magnum, making targeted sampling of the amygdala and hippocampus, preferred sites for detection, difficult (Nishimura et al. 2013).

All 14 blood samples from seropositive cows tested negative, unlike reported rates in Iraq (12.36%) and Colombia (13.5%) (Al-Gharban, Al-Eodawee & Al-Shabbani 2017; Correa-Castro, Gómez-Palacio & Pulido-Medellín 2021). This discrepancy may be attributed to the possibility that *N. caninum* infection was in a latent phase at the time of sampling, characterised by the predominance of tissue-bound bradyzoites and the absence of circulating tachyzoites. During such latent stages, the likelihood of detecting parasite DNA in peripheral blood is significantly reduced, which could explain the negative molecular results despite seropositivity.

This study provides a baseline for future investigations in Namibia. Targeting high-risk populations, such as aborted foetuses, over extended periods would enhance the likelihood of detecting *N. caninum* DNA.

## Conclusion

This study highlights the significant challenges in detecting *N. caninum* in Namibian cattle, primarily because of a low seroprevalence and environmental conditions that are inhospitable to oocyst survival. The arid climate and intense solar radiation prevalent in the region likely reduce the persistence of oocysts in the environment, thereby limiting opportunities for transmission. Furthermore, although farm dogs, stray dogs, and jackals, commonly found on freehold farms, are potential sources of infection, their actual contribution to the epidemiology of *N. caninum* in this setting remains uncertain.

Limitations such as insufficient sampling and a small sample size could also have impacted results. Future research should focus on high-risk groups like aborted foetuses and use larger, targeted sample sizes over time to improve detection. Moreover, faecal samples from farm dogs and jackals could be systematically collected and analysed to confirm the presence of *N. caninum* oocysts, thereby providing critical epidemiological evidence regarding their potential role as definitive hosts in the parasite’s transmission cycle. This study provides a foundational understanding of bovine neosporosis in Namibia’s arid ecosystem.
